# Assessment of validity evidence for the RobotiX robot assisted surgery simulator on advanced suturing tasks

**DOI:** 10.1186/s12893-020-00839-z

**Published:** 2020-08-12

**Authors:** Erik Leijte, Ivo de Blaauw, Camiel Rosman, Sanne M. B. I. Botden

**Affiliations:** 1 Department of Surgery, Radboud University Medical Center, Geert grooteplein 10 route 618, 6500HB Nijmegen, The Netherlands; 2 Department of Pediatric Surgery, Radboud University Medical Center, Geert grooteplein 10 route 618, 6500HB Nijmegen, The Netherlands

**Keywords:** Robotic surgery, Virtual reality simulation, Proficiency based training, Validation

## Abstract

**Background:**

Robot assisted surgery has expanded considerably in the past years. Compared to conventional open or laparoscopic surgery, virtual reality (VR) training is an essential component in learning robot assisted surgery. However, for tasks to be implemented in a curriculum, the levels of validity should be studied for proficiency-based training. Therefore, this study was aimed to assess the validity evidence of advanced suturing tasks on a robot assisted VR simulator.

**Method:**

Participants were voluntary recruited and divided in the robotic experienced, laparoscopic experienced or novice group, based on self-reported surgical experience. Subsequently, a questionnaire on a five-point Likert scale was completed to assess the content validity. Three component tasks of complex suturing were performed on the RobotiX simulator (Task1: tilted plane needle transfer, Task: 2 intracorporal suturing, Task 3: anastomosis needle transfer). Accordingly, the outcome of the parameters was used to assess construct validity between robotic experienced and novice participants. Composite scores (0–100) were calculated from the construct parameters and corresponding pass/fail scores with false positive (FP) and false negative (FN) percentages.

**Results:**

Fifteen robotic experienced, 26 laparoscopic experienced and 29 novices were recruited. Overall content validity outcomes were scored positively on the realism (mean 3.7), didactic value (mean 4.0) and usability (mean 4.2). Robotic experienced participants significantly outperformed novices and laparoscopic experienced participants on multiple parameters on all three tasks of complex suturing. Parameters showing construct validity mainly consisted of movement parameters, needle precision and task completion time. Calculated composite pass/fail scores between robotic experienced and novice participants resulted for Task 1 in 73/100 (FP 21%, FN 5%), Task 2 in 85/100 (FP 28%, FN 4%) and Task 3 in 64/100 (FP 49%, FN 22%).

**Conclusion:**

This study assessed the validity evidence on multiple levels of the three studied tasks. The participants score the RobotiX good on the content validity level. The composite pass/fail scores of Tasks 1 and 2 allow for proficiency-based training and could be implemented in a robot assisted surgery training curriculum.

## Background

Robot assisted surgery has been widely accepted during the past years and continues to grow which leads to more surgeons being trained in robot assisted surgery [1]. Training of robot assisted surgery is often compared to the training of an airline pilot, because both deal with complex technology and have very limited room for errors, which could result in severe complications. Therefore, these circumstances demand an extensive standardized training curriculum before a surgical trainee is fit to ‘fly’ [2].

There are multiple modalities used to learn robot assisted surgery [2, 3]. Proctoring often consists of an external expert providing direct supervision during surgery. Although this method has never proven its efficacy it is generally accepted that it allows for a safe and interactive learning. However, proctoring is expensive when required for a more extensive period [4]. Mentoring using a mentor console provides a safe collaboration between the trainee and a local experienced mentor [5]. Unfortunately, this method of training is limited in availability of an additional ‘mentor’ console and requires additional informed consent. Before proctoring or mentoring, simulation models are used to practice robotic skills or procedures in a safe environment. Simulation models primarily consist of virtual reality (VR) simulation, inanimate models and live animal or cadaveric training. While cadaveric training has the benefits of the realistic anatomy and the opportunity for procedural training, it remains costly and comes with ethical concerns [6, 7]. The training with inanimate models such as 3D-printed anatomical structures is a safe and realistic training method, but limited due to the requirement of training instruments and access to a live console. Therefore, the use of VR simulation is a widely accepted effective method to train robot assisted surgery from basic and advanced skills to procedural training [8]. The training with VR simulators is already a proven valuable tool for laparoscopic surgery and as a possible preoperative warm up [8–10].

However, for the use of VR simulation validation studies are required to determine the usefulness of training [11–13]. This allows for training aimed at improving proficiency [14]. Most validation studies on surgical robot simulators performed are aimed at *basic* surgical tasks and are assessed between novice and robot experienced participants [14]. Then again, the main advantage of robot assisted surgery is expected during *complex* tasks, which often require suturing skills and working in a small space. Therefore, laparoscopic participants are also a target group for learning robot assisted surgery besides novices [15]. The goal of this study is to collect the validity evidence according to Messick’s contemporary framework for advanced robot assisted tasks on the RobotiX robot assisted VR simulator and to establish a proficiency score [11, 16]. Besides, novice participants, we also take in account the performance of laparoscopic experienced participants.

## Methods

### Participants

Participants included in this study were voluntary recruited at the Radboud university medical center Nijmegen, the Netherlands and during the European Association of Urology congress Copenhagen 2018. To prevent influence of work fatigue, the simulations were conducted outside of the OR and only during the morning or afternoon. The subjects were dived in either of the three groups based on their self-reported surgical experience. Participants with a medical background and understanding of minimal invasive surgery but without clinical surgical experience were selected in the ‘novice group’ as a control group. Participants with clinical laparoscopic experience and without robot assisted experience were allotted to the ‘laparoscopic experience group’. The laparoscopic experienced group was included in this study because they are unexperienced with robot assisted surgery and most likely the next participants to learn robot assisted surgery and therefore, the target group. Finally, the robot assisted experienced participants with > 10 robot assisted clinical procedures experience were allotted to the ‘robotic experience group’.

### Questionnaire

For the content validity evidence, a previously used questionnaire was adapted for this study [17–19]. The questionnaire consisted of a section regarding the participants informed consent, demographic information and surgical experience and can be found in Supplemental 1. The second section was completed after performing the three tasks and consisted of multiple questions on a five-point Likert scale. These questions were divided in the domains; realism, didactic value and usability per task. With ‘1’ representing ‘in strong disagreement’, ‘3’ as a ‘neutral opinion’ and ‘5’ being in ‘strong agreement’ [20]. Outcomes of > 3.5 were considered as positive scores. The realism was assessed using questions concerning the perceived realism, grasper manipulation, tissue handling and on-screen response. The didactic value contained questions regarding the value to train inexperienced and experienced surgeons, and the value to assess the skills of a trainee. The usability was scored by the participants on the user-friendliness of the simulator interface and the appeal of the system to train for this task.

### Simulator and metrics

**Fig. 1:**
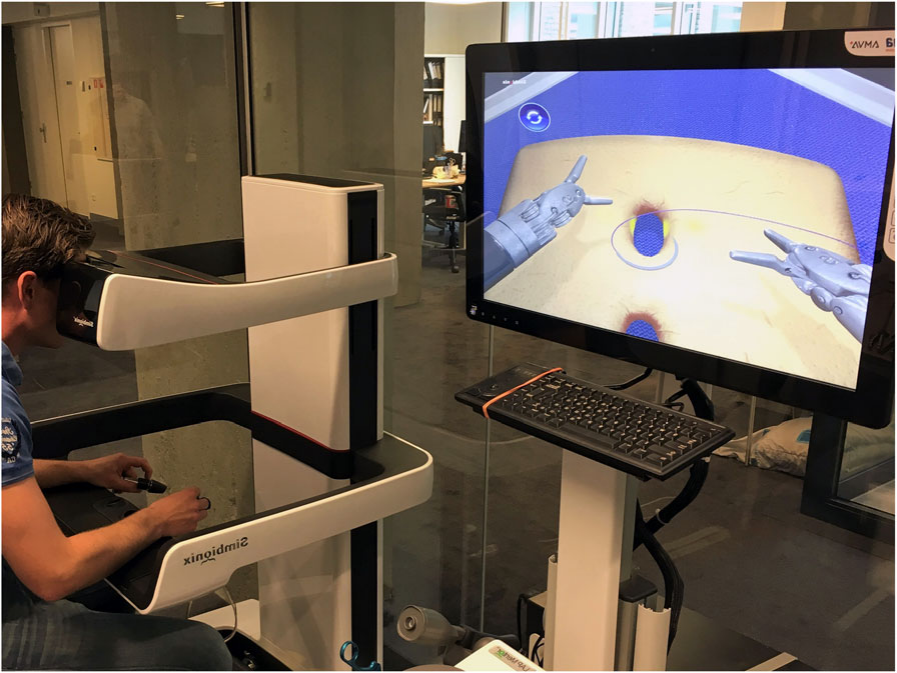
setup of the RobotiX Mentor VR simulator as used in this study

The standard supplied setup of the RobotiX Mentor (3D systems, Colorado, USA) platform was used in this study (Fig. 1). The system consisted of an operating tower containing the computer with screen and the console unit which functions as the workspace with the simulator master controls and the 3D viewer. The platform installation and user instruction were provided by 3D Systems to ensure correct use. The system is designed to mimic the da Vinci® Surgical System (Intuitive Surgical Inc., California, USA). This is done by the freehand controls, 3D view and similar ergonomic workspace setting which can be personally adjusted. The supplied software ‘Mentorlearn’ was used for tracking the performance parameters per participant. The software kept track of twelve to twenty-five parameters per tasks from which the most clinically significant ones were selected by experienced surgeons to be included in this study. The included parameters were accordingly divided in three domains consisting of; movement, safety and task specific parameters. The parameter definitions are stated in Table 1.

**Table 1:** parameter definitions of the selected clinically relevant parameters

Movement parameters	Definition
Path length (left/right)	Distance travelled by instrument in millimeter
Movements (left/right)	Number of movements made by instrument
Entrance and exit points	Total number of entrance and exit points
Distance scope and tissue	Average distance between scope and vaginal cuff in millimeter
**Safety parameters**	
Inaccurate punctures	Number of needle punctures not within relevant target mark
Suture breakage	Number of times the suture broke due to excessive force
Instrument collisions	Number of times instruments collided
Times out of view	Number of times the users instrument was held out of view
Dropped needles	Number of times a needle was dropped
Unnecessary piercing	Number of unnecessary piercing points
**Task specific parameters**	
Total time	Total time in seconds
Total errors	Total number of errors made
Needle precision	Percentage of needle punctures which are on relevant target mark
Needle passages	Total number of needle passages made
Precise needle passages	Number of needle passages on relevant mark
Total knots	Total number of knots made
Surgeon knots	Total number of surgical knots

### Tasks

The tasks selected for this study were based on their representation of skills required during complex suturing surgery. This is where the most advantage of the robotic assistance is to be expected compared to conventional minimally invasive surgery.

**Fig. 2:**
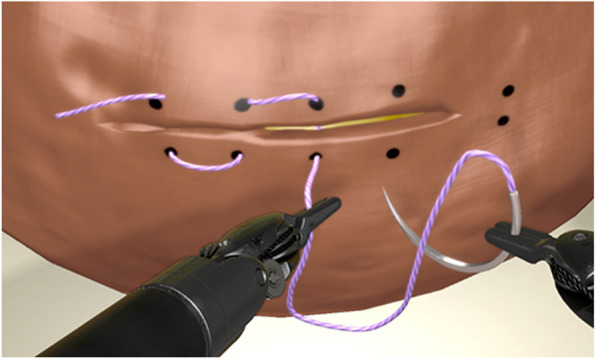
Screenshot of Task 1 Railroad track (tilted plane needle transfer), figure provided by 3D systems

Task 1: Railroad track (Fig. 2) is a needle transfer task in a tilted plane without knot tying. The supplied needle and thread had to be transferred through multiple dots in a matrass pattern. To complete the task the needle had to be anchored in the virtual ball.

**Fig. 3:**
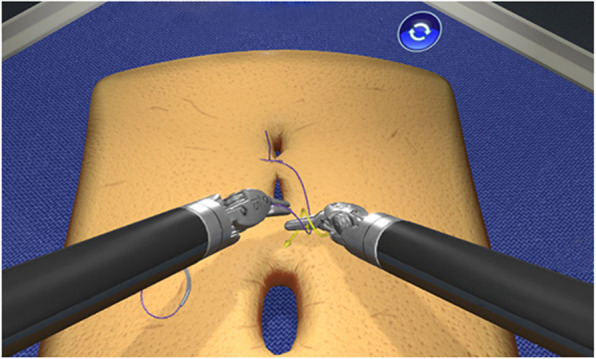
Screenshot of Task 2 Intracorporal suturing, figure provided by 3D systems

Task 2: Intracorporal suturing (Fig. 3) is a standard suturing task where two surgical knots had to be placed on a virtual suturing pad. The system gave instructions during the tasks which was finished when two knots have been placed.

**Fig. 4:**
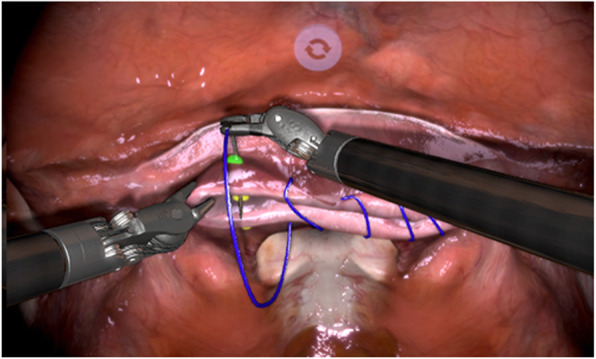
Screenshot of Task 3 Vaginal cuff closure (anastomosis needle transfer), figure provided by 3D systems

Task 3: Vaginal cuff closure (Fig. 4) simulates an anastomosis needle transfer task without knot tying. The task was performed with a barbed wire suture which is used to close a vaginal cuff (after hysterectomy) with guidance from highlighted dots. Once the required transfers were made and the suture was cut, the task was completed.

### Protocol

Upon entering, the study participants completed the first part of the questionnaire regarding their demographics and surgical experience. The ‘response validity’ was maintained by having a single researcher giving the handling, system instructions and explaining the written ‘Mentorlearn’ task. To attain familiarity with the system, participants first performed two basic tasks concerning the wristed capability and tissue handling. Subsequently the three selected suturing tasks were performed. A maximum of 20 min was given for the completion of the tasks and performance outcomes were saved by the ‘Mentorlearn’ software. At completion of the performed tasks the participant completed the remainder of the questionnaire on the realism, didactic value and usability per task, to assess the ‘content source of validity’. This assessment is mainly based on the opinion of the robotic experienced participants, because they have the clinical experience. However, the novices and laparoscopic experienced participants are the possible future trainees for robot assisted surgery and were, therefore, included. The performance scores of each group were used to assess the ‘relation to other variables validity’ by comparing performance outcomes for parameters being statistically significantly different and thus showing construct validity. Accordingly, a composite score was calculated with the construct parameters to determine a pass/fail score for the ‘consequence of the test validity’.

### Statistical analysis

Data analysis was performed using the Statistical package for social sciences (SPSS) version 25 (IBM Corp., New York, USA). All *P*-values < 0.05 were considered statistically significant.

The content and relation to other variables validity were analyzed with the outcomes from the questionnaire and the performance scores using independent t-test between each group after testing for normal distribution. Statistically significant different performance outcomes between novice and robotic experienced participants were included for the composite score calculation. Parameters resulting in ‘better’ performance for the novice group were excluded for the composite score calculation. The composite score was calculated from the mean value of the selected parameters after linear normalization ranging from 0 to 100 with the latter being the highest score.

Consequence validity was analyzed by using the calculated composite score for a pass/fail cutoff value, which was determined by the contrasting groups method. For this method the model by Jorgensson et al. was used and adapted to incorporate the mean score and standard deviation of three groups to calculate the optimal pass/fail scores [21]. Additionally, this model calculates the theoretical false positive and false negatives, which can be used as an addition to the absolute false positives and false negatives, because these are prone to be unreliable for small sample sizes and outliers [21].

## Results

### Demographics

A total of 70 participants were included of which 29 novices, 26 laparoscopic experienced and 15 robotic experienced participants with the characteristics shown in Table 2. The novice group consisted of medical students with a mean age of 24 years without any laparoscopic or robot assisted experience. The laparoscopic group contained seventeen residents in training and nine specialized surgeons from surgical, urologic and gynecologic specialties. The mean age was 35 years and 92% right-handed dexterity. The robotic experienced group consisted of five residents in their fourth till sixth year of specialty training and ten specialized surgeons. Robotic experience ranged from 0 to > 50 basic procedures completed by seven participants and > 50 basic procedures completed by eight participants. The number of advanced procedures ranged from 0 to 50 for eight participants with the remaining seven participants having completed > 50 advanced procedures. Mean age and dexterity in the robotic experienced group was 43 years and 73% right-handedness respectively.

**Table 2:** Demographics

	Robotic experience	Laparoscopic experience	Novices
N	15	26	29
Mean age (SD)	43 (10.8)	35 (5.5)	24 (2.4)
Sex (male/female)	14/1	15/11	12/17
Dexterity (right/left/ambidextrous)	11/0/3	24/2/0	25/4/0
Surgical skill level			
Medical student	–	–	29
Resident in training			
1–2 years	0	2	–
3–4 years	1	11	–
5–6 years	4	4	–
Surgeon	10	9	–
Specialty			
None	–	–	29
Surgery	3	8	–
Urology	11	4	–
Gynecology	1	10	–
Pediatric	0	4	–
Overall laparoscopic experience (years)			
0	1	0	29
< 1	0	1	–
1–5	5	18	–
5–10	1	2	–
> 10	8	5	–
Robot assisted experience			
Basic procedures			
0	1	25	29
< 10	2	1	–
11–30	4	0	–
31–50	0	0	–
> 50	8	0	–
Advanced procedures			
0	2	26	29
1–20	6	–	–
21–50	0	–	–
> 50	7	–	–

*SD* standard deviation. Procedures with intracorporal suturing were considered advanced

### Content (realism, didactic value and usability)

The opinion values of the three tasks are shown in Table 3. The overall score for the realism, didactic value and usability was rated positively. The robotic experienced participants scored the usability of the system significantly lower than the novice group for all tasks (*p*-values 0.007, 0.002 and 0.048 respectively). However, the lowest mean usability score by the robotic experienced participants was 3.9, which is still rated good. The realism was scored lowest by the robotic experienced participants on all tasks (3.5, 3.4 and 3.5 respectively) resulting in a neutral to moderate positive opinion on the realism. The lowest realism scores from the robotic experienced participants were found at the behavior of sutures running through the tissue of Task 1 and 3 (mean 3.3 and 3.1) and the thread behavior at Task 2 (mean 3.1). The highest mean realism scores from the robotic experienced were found for the realism to mimic needle transfer at Task 1 (mean 3.7) and the realistic on-screen response during Task 2 and 3 (both mean 3.9). The laparoscopic participants scored the realism of Task 3 statistically significantly higher than the robotic experienced (4.1 versus 3.5, *p* = 0.009) and novice group (4.1 versus 3.6, *p* = 0.005). This is also seen at the realism sub questions ‘realism to mimic vaginal cuff closure’ (laparoscopic 4.2 versus robot 3.5 and novices 3.6, *p* = 0.018 and *p* = 0.024) and suture behavior (laparoscopic 3.9 versus robot 3.1 and novices 3.4, *p* = 0.006 and *p* = 0.016). This indicates a disagreement in realism perception between the laparoscopic participants and the remaining groups. All three groups agreed concerning the didactic value, scoring it positively for all tasks (overall means of 3.9, 4.1 and 4.0 respectively). The specific lowest didactic value scores by the robotic experienced participants were found for the didactic value to train experienced surgeons on all tasks (mean 3.8, 3.7 and 3.6, respectively).

**Table 3:** Mean opinion scores (standard deviation) for the three domains of the questionnaire

	Robotic experience	Laparoscopic experience	Novices	Total group	***p***-values		
**Task 1**					R vs N	L vs N	R vs L
Realism	3.5 (0.7)	3.5 (0.6)	3.7 (0.5)	3.6 (0.6)	0.230	0.106	0.853
Didactic value	4.0 (0.9)	3.7 (1.0)	4.0 (0.5)	3.9 (0.8)	0.892	0.156	0.354
Usability	3.9 (0.6)	4.2 (0.6)	4.4 (0.5)	4.1 (0.6)	**0.007**	0.195	0.161
**Task 2**							
Realism	3.4 (0.8)	3.8 (0.5)	3.8 (0.5)	3.7 (0.6)	0.078	0.975	0.082
Didactic value	4.0 (0.7)	4.2 (0.5)	4.2 (0.4)	4.1 (0.6)	0.274	0.923	0.360
Usability	3.9 (0.6)	4.3 (0.6)	4.5 (0.5)	4.2 (0.6)	**0.002**	0.131	0.094
**Task 3**							
Realism	3.5 (0.7)	4.1 (0.5)	3.6 (0.4)	3.7 (0.6)	0.470	**0.005**	**0.009**
Didactic value	3.9 (0.8)	4.1 (0.8)	4.1 (0.6)	4.0 (0.7)	0.386	0.801	0.564
Usability	4.0 (0.7)	4.2 (0.6)	4.4 (0.5)	4.2 (0.6)	**0.048**	0.227	0.335

Data in this table represents mean scores and standard deviation. Statistical differences were calculated using independent t-test. A *p*-value of < 0.05 was considered statistically significant (displayed in bold)

### Relation with other variables (construct)

#### Task 1

The mean performance score of Task 1 are presented in Table 4. Statistically significant differences in performance outcomes between the robotic experienced versus novices and laparoscopic group was shown for all the included movement parameters, as well as the ‘inaccurate punctures’, ‘instrument collisions’, ‘needle precision’ and ‘total time’ parameters (*p*-values < 0.001–0.014). The laparoscopic experienced participants only scored significantly better than the novice participants for the ‘total time’ parameter (475 s versus 597 s, *p* = 0.047 respectively).

**Table 4:** Mean (SD) performance outcomes per group on Task 1

Task 1: Railroad track	Robotic experience	Laparoscopic experience	Novices	***P***-values		
**Movements**	*N* = 15	*N* = 26	*N* = 29	R vs N	L vs N	R vs L
Path length left	1655 (478)	3469 (1577)	4157 (1870)	**< 0.001**	0.148	**< 0.001**
Path length right	2196 (771)	3879 (2144)	3885 (1720)	**0.001**	0.991	**0.001**
Movements left	168 (57)	342 (159)	422 (176)	**< 0.001**	0.085	**< 0.001**
Movements right	234 (76)	394 (191)	425 (170)	**< 0.001**	0.533	**0.001**
**Safety**						
Inaccurate punctures	5.5 (3.1)	10.9 (7.3)	13.4 (9.4)	**< 0.001**	0.274	**0.002**
Instrument collisions	4.0 (3.9)	13.9 (13.9)	15.9 (10.5)	**< 0.001**	0.544	**0.002**
Times out of view	1.3 (1.0)	2.4 (4.0)	1.7 (2.1)	0.663	0.435	0.581
**Task specific**						
Needle precision	63 (15)	48 (20)	47 (19)	**0.007**	0.926	**0.014**
Total errors	13.9 (12.1)	11.1 (5.5)	11.3 (3.3)	0.452	0.896	0.322
Total time	265 (92)	475 (219)	597 (225)	**< 0.001**	**0.047**	**< 0.001**

Data in this table represents mean performance scores and standard deviation. *R* Robotic experienced, *L* Laparoscopic experienced, *N* Novices. *P*-values were calculated using independent t-test, values of < 0.05 were considered statistically significant (displayed in bold).

#### Task 2

In the second task the robotic experienced group performed statistically significantly higher than the novice group on the parameters; ‘entrance and exit points’, ‘dropped needles’, ‘unnecessary needle piercing’, ‘suture breakage’, ‘needle precision’ and ‘total time’ as shown in Table 5. Similar results were found when comparing the robotic with the laparoscopic experienced group regarding ‘unnecessary needle piercing’, ‘suture breakage’, ‘needle precision’ and ‘total time’ parameters. Although, there was a difference in the parameter ‘needle out of view’ in favor of the robotic experienced participants, this was not statistically significantly different. The ‘total knots’ and ‘surgeon knots’ were not significantly different between the groups, although, the system knot scoring was strict and did not allow for knot variations. The laparoscopic experienced participants significantly outperformed the novice group on the ‘dropped needles’, ‘unnecessary piercing points’ and ‘total time’ parameters (*p*-values 0.039, 0.036 and < 0.001 respectively). At Task 2 a technical error occurred resulting in loss of performance data of one novice and one robotic experienced participant.

**Table 5:** Mean (SD) performance outcomes per group on Task 2

Task 2: intracorporal suturing	Robotic experience	Laparoscopic experience	Novices	***P***-values		
**Movements**	*n* = 14	*n* = 26	*n* = 28	R vs N	L vs N	R vs L
Entrance and exits points	5.7 (2.1)	7.7 (4.6)	11.4 (8.8)	**0.003**	0.064	0.075
**Safety**						
Dropped needles	3.6 (3.5)	5.8 (6.8)	10.8 (10.4)	**0.002**	**0.039**	0.268
Unnecessary piercing	2.6 (2.6)	6.3 (5.0)	10.4 (8.4)	**< 0.001**	**0.036**	**0.004**
Needle outside of view	3.3 (4.8)	6.2 (9.9)	8.1 (11.2)	0.136	0.534	0.301
Suture breakage	0.0 (0.0)	0.3 (0.5)	0.6 (1.2)	**0.007**	0.133	**0.016**
**Task specific**						
Total knots	1.5 (0.9)	1.3 (0.7)	1.1 (0.4)	0.123	0.324	0.373
Surgeon knots	0.6 (1.0)	0.6 (0.8)	0.8 (0.6)	0.473	0.281	0.985
Needle precision	96 (8)	79 (30)	90 (13)	**0.042**	0.101	**0.008**
Total time	171 (77)	277 (172)	546 (328)	**< 0.001**	**< 0.001**	**0.011**

Data in this table represents mean performance scores and standard deviation. *R* Robotic experienced, *L* Laparoscopic experienced, *N* Novices. *P*-values were calculated using independent t-test, values of < 0.05 were considered statistically significant (displayed in bold)

#### Task 3

Table 6 shows the mean performance outcomes for Task 3. Statistically significant better performance scores of the robotic experienced compared to the novice group were found for the following four parameters; ‘path length left’, ‘instruments collisions’, ‘precise needle passages’ and ‘total time’ (*p*-values 0.015, 0.001, 0.032 and 0.024 respectively). Interestingly, the laparoscopic experienced group was significantly outperformed by the robotic group on six parameters; ‘path length left’ (*p* < 0.001), ‘movements left’ (*p* = 0.018), ‘entrance and exit points’ (*p* = 0.013), ‘instruments collisions’ (*p* = 0.002), ‘needle passages’ (*p* = 0.013) and ‘total time’ (*p* = 0.040). Although, some statistical differences were found in the previous tasks for laparoscopic experienced versus novice participants, none were apparent in this task. The robotic experienced participants had their instruments more often out of view than the novice participants (32 versus 16 times, *p* = 0.030). Therefore, the ‘times out of view’ parameter could not be included in the composite score. Additionally, the robotic experienced group worked significantly closer on the target tissue than the novice and laparoscopic groups as is seen in the ‘distance scope and tissue’ parameter (94 mm versus 120 mm and 116 mm. *p* < 0.001 and *p* = 0.001 respectively). There were less unnecessary needle piercings in the robotic experienced group compared to both other groups (mean 10.8 versus 14.4 and 13.1), However, this was not statistically significant.

**Table 6:** Mean (SD) performance outcomes per group on Task 3

Task 3: Vaginal cuff closure	Robotic experience	Laparoscopic experience	Novices	***p***-values		
**Movements**	*n* = 15	*n* = 26	*n* = 29	R vs N	L vs N	R vs L
Path length left	3813 (1508)	6862 (3261)	5544 (2397)	**0.015**	0.091	**< 0.001**
Path length right	4784 (1851)	5941 (2965)	5377 (2937)	0.482	0.482	0.133
Movements left	344 (127)	494 (212)	438 (164)	0.060	0.279	**0.018**
Movements right	386 (156)	501 (229)	450 (182)	0.258	0.367	0.065
Entrance and exit points	28 (4)	32 (7)	31 (5)	0.105	0.291	**0.013**
Distance scope and tissue	94 (20)	116 (10)	120 (1)	**< 0.001**	0.056	**0.001**
**Safety**						
Instrument collisions	9 (11)	32 (32)	24 (19)	**0.001**	0.280	**0.002**
Times out of view	32 (34)	16 (11)	10 (16)	**0.030**	0.148	0.088
Unnecessary piercing	10.8 (8.4)	14.4 (11.1)	13.1 (10.1)	0.460	0.647	0.284
Suture breakage	0.0 (0.0)	0.0 (0.2)	0.0 (0.2)	0.479	0.939	0.455
**Task specific**						
Precise needle passages	10.3 (3.0)	12.0 (3.9)	12.4 (3.0)	**0.032**	0.661	0.153
Needle passages	14.1 (1.8)	16.1 (3.3)	15.3 (2.5)	0.105	0.291	**0.013**
Total time	423 (180)	580 (250)	573 (210)	**0.024**	0.916	**0.040**

Data in this table represents mean performance scores and standard deviation. *R* Robotic experienced. *L* Laparoscopic experienced. *N* Novices. *P*-values were calculated using independent t-test. Values of < 0.05 were considered statistically significant (displayed in bold)

### Sub-expert analysis

In order to determine the influence of higher robotic assisted surgical experience, a sub group of robotic experienced participants with > 50 advanced procedures (*n* = 7) was used. This sub-expert group resulted in construct validity for the same parameters as the robotic experience group compared to the novice group and was therefore not used in further analysis.

### Consequences (composite score and contrasting group)

Calculation of the composite score per task led to a composite score for Task 1 consisting of the parameters; ‘path length left’, ‘path length right’, ‘movements left’, ‘movements right’, ‘inaccurate punctures’, ‘instrument collisions’, ‘needle precision’ and ‘total time’ parameters. The composite score of Task 2 was calculated with the parameters; ‘suture breakage’, ‘entrance and exit points’, ‘dropped needles’, ‘unnecessary needle piercing’, and ‘total time’. Task 3 consists of the parameters; ‘path length left’, ‘instruments collisions’, ‘precise needle passages’ and ‘total time’. The ‘times out of view’ parameters and ‘distance scope and tissue’ parameters were not included in the composite score because the novice group outperformed the robotic group on the ‘times out of view’ parameter.

**Fig. 5:**
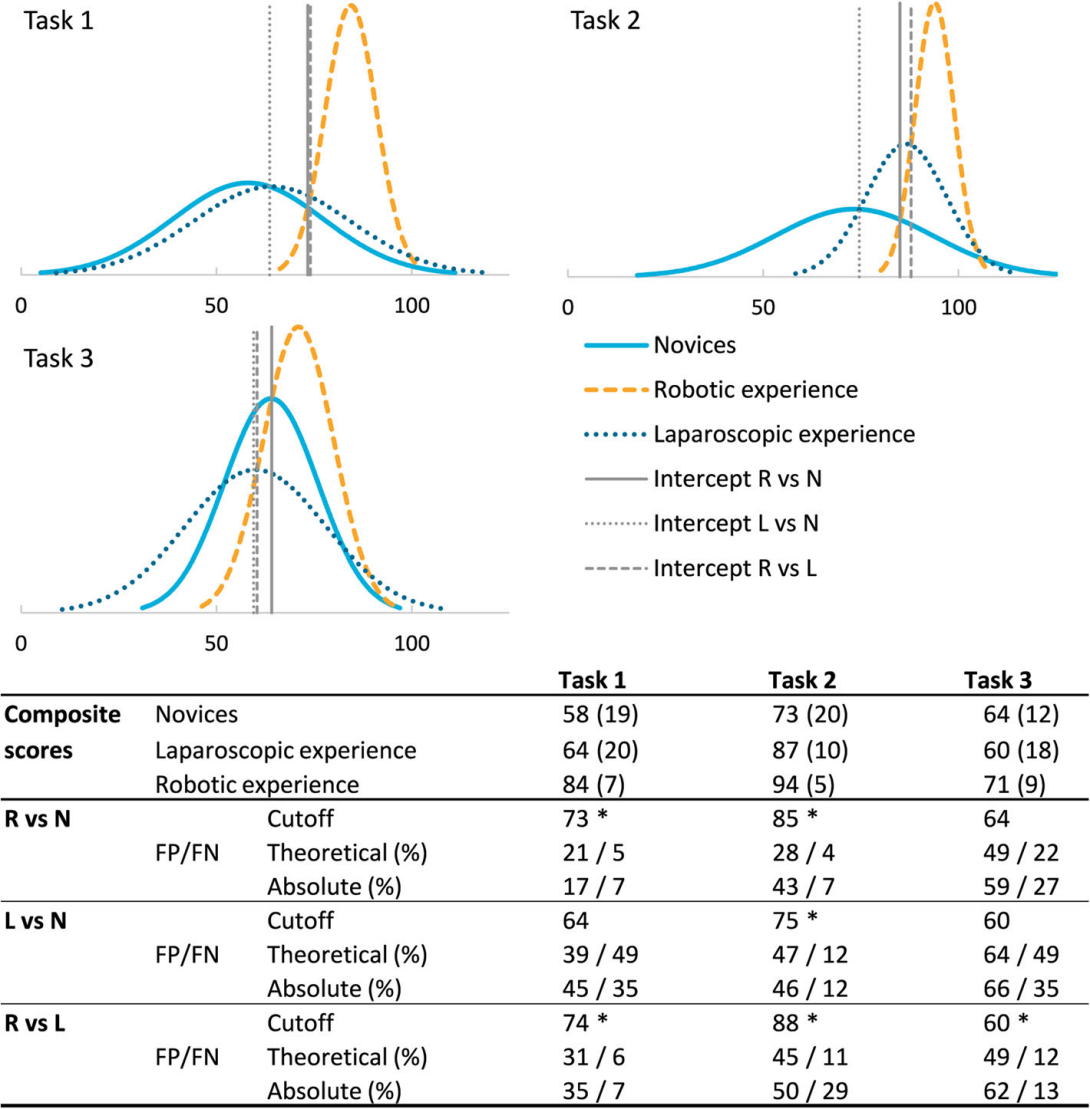
Mean composite score outcome and contrasting group analysis results of Task 1–3 with the corresponding theoretical and absolute false positive (FP) and false negative (FN) percentages. Data in this table represents mean composite outcomes with standard deviation. Cutoff values were calculated using the contrasting groups method. R = Robotic experienced. L = Laparoscopic experienced. N = Novices. FP = false positive percentage, FN = false negative percentage. * indicates a *p*-value < 0.05 between the corresponding groups based on the mean composite score

The results for the contrasting group analysis using the composite scores are shown in Fig. 5. The cutoff values, theoretical false positive and false negative percentages were calculated between all three groups. The lowest theoretical false positive/false negative percentage was found for Task 1 at a cutoff value of 73 and 74 between novice and laparoscopic participants versus robotic experienced (21/5% and 31/6%). The mean composite score of Task 2 shows a gradual increase between the experience groups. The cutoff value between novice and laparoscopic participants was found at 85 and 88 with a false positive/false negative percentage of 28/4% and 45/11%. The cutoff score for Task 3 shows the lowest discriminative ability between the novices and robotic experienced with 49% false positives and 22% false negatives. A sub analysis was performed for each task by weighing the included parameters in a best/worst case scenario, however, this did not result in a significantly better discriminative ability.

## Discussion

In this study the levels of validity evidence were assessed according to Messick’s framework [11, 16] for three suturing tasks on the RobotiX VR simulator. Results show a positive content validity evidence, with room for improvement regarding the realism of all three tasks. The usability was scored good to excellent particularly by the laparoscopic (target) group (means 4.2–4.3). Additionally, the didactic value was scored good by the robotic experienced participants for all three tasks (means 3.9–4.0). The relationship to other variables and the consequence evidence validity resulted in a usable composite score with an accompanying pass/fail score for the tilted plane needle transfer (Task 1) and intracorporal suturing (Task 2) tasks. These scores allow for valid proficiency-based training which can be implemented in a robot assisted curriculum to assess the skills of a trainee. The third task (anastomosis needle transfer) seemed to be either too difficult for our expert group or was too strict in the assessment parameters to result in a valid composite score (Fig. 5). The laparoscopic experienced were unable to show adequate discriminative ability from the novices and robotic experienced group based on the composite score (Fig. 5). Although, the laparoscopic experienced were able to show some construct parameters and higher average composite score outcomes versus the novice group for Task 1 and 2.

Previous validation studies were performed regarding the validity of the RobotiX simulator [22–28]. However, only limited studies were performed using the contemporary framework of validity [24]. The manuscript by Hovgaard et al. recently studied the Vaginal cuff closure task (Task 3 in this study) and found similar parameter outcomes as this study [24]. Construct between novices and robotic experienced participants was found in both studies for the ‘path length’, ‘instrument collisions’ and ‘total time’ parameters. Although our study also found construct for the ‘precise needle passages’ parameter, it was not shown for the ‘unnecessary piercing points’ parameter, as Hovgaard et al. found. Interestingly, they reported that robotic experienced participants used the camera functionality significantly more, therefore working closer on the target area and scoring significantly higher on the out of view parameters [24]. This effect was also shown in the current study, with a statistically significant difference in the ‘distance scope and tissue’ and ‘times out of view’ parameters between the novice and robotic experienced group. Consequently, this makes the ‘out of view’ parameter unfit for the proficiency composition if not corrected for the distance. However, when learning robot assisted surgery it is important in terms of safety to keep instruments in view at all time, due to the lack of haptic feedback. This may also indicate the potential pitfalls of using experienced robotic surgeons. The calculated pass/fail score by Hovgaard et al. was based on participants fifth and sixth repetition of a learning curve which showed an absolute false positive and false negative percentage of 36 and 27% respectively. This study shows a similar false negative percentage (27%) but is unable to reproduce the false positive percentage (36% in this study). Possible differences are the parameters included for the composite score calculation, number of participants (11 novices and 11 robotic experienced versus 15 robotic experienced and 19 novices in this study) and the number of repetitions that participants performed.

The three main strengths of this study are the relatively high number of participants (*n* = 70), the inclusion of the laparoscopic participants as the target group to learn robot assisted surgery and the calculation of composite scores for multiple tasks. However, there are some limitations to this study as well. The novice group scored the usability significantly higher compared to robotic experienced for all tasks. Although, both groups were highly positive, this result shows a possible influence by the novelty of this technique for the novice group. Therefore, positive conclusions on base of the novice group are limited. The performance results showed a valid composite pass/fail score for Task 1 and 2 however, for Task 3 the composite pass/fail score resulted in a higher percentage of false positives and false negatives which indicates a poor sensitivity and specificity. This is most likely because the construct validity was only shown for four out of twenty-five parameters provided by the simulator. Interestingly, in this specific group, there were more statistically significant differences in the parameters of the laparoscopic versus the robotic experienced group than for the novice versus robotic group (six versus four parameters). This could be due to the inexperience of the novice group, which caused more careful handling and therefore, better performance. Concerning the intracorporal suturing (Task 2), the main goal was the correct knot placement, although this study could not show construct validity for any knot specific parameter. Also, during Task 2 multiple participants noticed errors with the simulated suture itself in this task, which led to the system scoring the tied knot as a single wrap where a double was placed. This limitation causes the calculated pass/fail score to be unable to score a trainee on the correctness of the knot. Results from all three tasks showed limited parameters with construct despite the wide variety of parameters available. This limitation in construct is also shown in previous studies [24, 28]. Therefore, a sub-expert analysis was performed (not shown) to assess increase of construct parameters using only more experienced robotic participants. However, this resulted in no additional parameters establishing construct validity.

Corresponding to the training of airline pilots, a training curriculum for robot assisted surgery should be composed of multiple modalities from which VR training is a single component [2]. Next should be the implementation of the tasks for proficiency-based training in a specific curriculum, in which the pass/fail limit should be reached before using other methods such as proctoring. Complemented by other training modalities, the proficiency-based VR training can be used to individually train component steps of specific procedures. These component steps should be validated in other simulation models to assess the transfer of skills.

## Conclusion

This study shows evidence of validity on the response, content relation to other variables and consequence levels for three suturing tasks on the RobotiX robot assisted simulator. The calculated composite pass/fail scores can be used for proficiency-based training with adequate discriminative power between novice and robotic experience in the tilted plane needle transfer and intracorporal suturing tasks. This can be implemented for trainees with or without laparoscopic experience as a proficiency goal in a robot assisted surgery training curriculum, supporting optimal training before starting with patient related robot assisted surgery.

## Supplementary information

**Additional file 1.** Questionnaire used in this study.

**Additional file 2.**

## Data Availability

The datasets used and analyzed during the current study are available as an additional supporting file.

## Abbreviations

VR: Virtual reality
FP: False positive
FN: False negative
