# Supplementary material for: Assessment of validity evidence for the RobotiX robot assisted surgery simulator on advanced suturing tasks
Source: BMC Surg. 2020 Aug 12;20:183. doi: 10.1186/s12893-020-00839-z (PMC7430880; doi:10.1186/s12893-020-00839-z)
Supplement: Supplementary file 1 — Additional file 1. Questionnaire used in this study. [file 12893_2020_839_MOESM1_ESM.docx]

**Supplemental 1:** Questionnaire used in this study

***
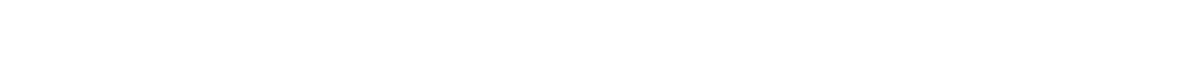
***

**Study background**

The aim of this study is to assess the face and content validity of several modules of RobotiX virtual reality robot simulator. This questionnaire aims to gather information about your surgical and laparoscopic experience and training and obtain your opinion on the level of realism, didactic value and usability. This research is done by Radboud University Medical Center.

**Personal Information & Informed consent**

Name: …………………………………………………………………………………… Male / Female

Hospital: ……………………………………………………………………………………

City: ……………………………………………………………………………………

Country: ……………………………………………………………………………………

E-­mail: ……………………………………………………………………………………

Age: ……… years

My dexterity is: Right handed

Left handed

Ambidextrous

I take part in this study voluntarily.

I was sufficiently informed in advance about the aim of the study and the contributions and activities that are asked from me within this study.

I am aware that the study in which I participate is a part of research. The researchers have my consent to use the anonymized results of the questionnaires and simulator exercises I take part in within the scope of this study for publications and presentations about the study.

Location (city & country): ………………………………………………………………

Date: …… / …… / 20…

Signature:

………………………………


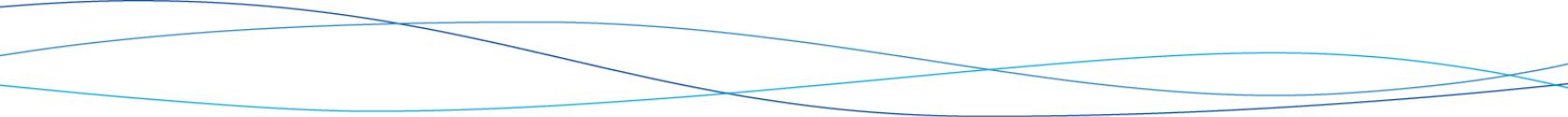
**Thank you for your participation!**

1

***
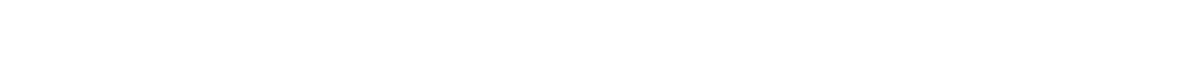
*Questionnaire – part 1: surgical experience & laparoscopy training**

1. What is your surgical skills level?

- Specialized surgeon
- Resident in training, in training year: 1 2 3 4 5 6
- Other: ………………………………

1. My specialty (to be) is (please tick the appropriate box):

| General Surgery | MIS | Abdominal Surgery | Oncologic Surgery | Vascular Surgery | Trauma Surgery | Pediatric Surgery | Thoracic surgery | Other: |
| --- | --- | --- | --- | --- | --- | --- | --- | --- |
|  |  |  |  |  | s |  |  | . |

1. I am practicing laparoscopy for approximately (please tick the appropriate box):

| 0 years | ≤1 year | 1-5 years | 5-10 years | ≥10 years |
| --- | --- | --- | --- | --- |
|  |  |  |  |  |

1. How many basic **laparoscopic** procedures (cholecystectomy & appendectomy) have you

performed in total (please tick the appropriate box)?

| 0 | ≤ 10 | 11-30 | 31-50 | 51-100 | ≥100 |
| --- | --- | --- | --- | --- | --- |
|  |  |  |  |  |  |

1. How many advanced **laparoscopic** procedures, including incorporeal suturing (e.g. fundoplication, bariatrics, esophageal atresia repaid etc.) have you performed in total (please tick the appropriate box)?

| 0 | ≤ 5 | 6-10 | 11-20 | 21-50 | ≥50 |
| --- | --- | --- | --- | --- | --- |
|  |  |  |  |  |  |

1. How many basic **robot assisted** procedures (cholecystectomy & appendectomy have you performed in total (please tick the appropriate box)?

| 0 | ≤ 10 | 11-30 | 31-50 | 51-100 | ≥100 |
| --- | --- | --- | --- | --- | --- |
|  |  |  |  |  |  |

1. How many advanced **robots assisted** procedures, including incorporeal suturing (e.g. fundoplication, bariatrics, oesophageal atresia repair, etc.) have you performed in total? (please tick the appropriate box)

| 0 | ≤ 5 | 6-10 | 11-20 | 21-50 | ≥50 |
| --- | --- | --- | --- | --- | --- |
|  |  |  |  |  |  |

1. Do you have any experience with basic or advanced skills training on simulators for **laparoscopy** or **robot** **assisted** simulators (please tick the appropriate box)?

- No (continue with question 9)
- Yes, please complete the following table (multiple answers are possible)

|  | Laparoscopy basic | Laparoscopy suturing/Advanced | Robot  basic | Robot  suturing/advanced |
| --- | --- | --- | --- | --- |
| Virtual reality simulators |  |  |  |  |
| Augmented reality simulators |  |  |  |  |
| Box trainers |  |  |  |  |
| Other |  |  |  |  |

- Tick the boxes that are applicable, more than one is allowed.

1. On which **virtual or augmented** reality simulator systems did you practice the following skills? (please complete the following table, multiple answers possible)

|  | Laparoscopy basic | Laparoscopy suturing/advanced | Robot  basic | Robot  suturing/advanced |
| --- | --- | --- | --- | --- |
| Simbionix Lap Mentor |  |  |  |  |
| SimSurgery SEP |  |  |  |  |
| Surgical Science LapSim |  |  |  |  |
| Mentice/Xitact Procedicus MIST-VR |  |  |  |  |
| CAE Healthcare LapVR (Immersion LapVR) |  |  |  |  |
| CAE Healthcare ProMIS (Haptica ProMIS) |  |  |  |  |
| EoSim (Eosurgical) |  |  |  |  |
| Da Vinci VR simulator |  |  |  |  |
| Other: ………………… |  |  |  |  |
| Don’t remember |  |  |  |  |

**This is the end of the first part of this questionnaire.**

**The next questions are to be completed after performing the tasks on the simulator.**

**Questionnaire – part 3: opinion on the RobotiX VR Robot simulator**

Please give your opinion on the properties of the specific aspects of the tasks on of this simulator. Indicate to what degree you agree with the following statements. There are no right or wrong answers.

**Task 1: Railroad track**

Please rate the following statements on a scale of 1 to 5, meaning:

**1**: Strongly disagree **2**: Disagree **3**: Neutral **4**: Agree **5**: Strongly agree

**Realism**

**Rate on a scale of 1 (strongly disagree) to 5 (strongly agree):**

|  | 1 | 2 | 3 | 4 | 5 | N/A |
| --- | --- | --- | --- | --- | --- | --- |
| The on­screen response of the tools matched my actions |  |  |  |  |  |  |
| The physical effort to manipulate the graspers was sufficiently realistic |  |  |  |  |  |  |
| The representation of this task to mimic needle transfer in the clinical setting was sufficiently realistic |  |  |  |  |  |  |
| The behavior of the tissue was sufficiently realistic |  |  |  |  |  |  |
| The behavior of sutures running through the tissue was sufficiently realistic |  |  |  |  |  |  |

**The didactic value**

**Rate on a scale of 1 (strongly disagree) to 5 (strongly agree):**

|  | 1 | 2 | 3 | 4 | 5 | N/A |
| --- | --- | --- | --- | --- | --- | --- |
| This module is valuable to train *inexperienced* surgeons with regards to *robot assisted* procedures |  |  |  |  |  |  |
| This module is valuable to train *experienced* surgeons with regards to *robot assisted* procedures |  |  |  |  |  |  |
| This module is valuable to assess the skills of a trainee |  |  |  |  |  |  |

**The usability of the simulator**

**Rate on a scale of 1 (strongly disagree) to 5 (strongly agree):**

|  | 1 | 2 | 3 | 4 | 5 | N/A |
| --- | --- | --- | --- | --- | --- | --- |
| The instructions on how to use the simulator by the research assistant were clear |  |  |  |  |  |  |
| The simulator interface (its on-screen design) is user-friendly |  |  |  |  |  |  |
| The RobotiX VR simulator is an appealing training tool for this task |  |  |  |  |  |  |

**Task 2: Vertical defect suturing**

Please rate the following statements on a scale of 1 to 5, meaning:

**1**: Strongly disagree **2**: Disagree **3**: Neutral **4**: Agree **5**: Strongly agree

**Realism**

**Rate on a scale of 1 (strongly disagree) to 5 (strongly agree):**

|  | 1 | 2 | 3 | 4 | 5 | N/A |
| --- | --- | --- | --- | --- | --- | --- |
| The on­screen response of the tools matched my actions |  |  |  |  |  |  |
| The physical effort to manipulate the graspers was sufficiently realistic |  |  |  |  |  |  |
| The representation of this task to mimic suturing in the clinical setting was sufficiently realistic |  |  |  |  |  |  |
| The behavior of the tissue was sufficiently realistic |  |  |  |  |  |  |
| The behavior of sutures running through the tissue was sufficiently realistic |  |  |  |  |  |  |
| The behavior of the thread in the knot tying was sufficiently realistic |  |  |  |  |  |  |

**The didactic value**

**Rate on a scale of 1 (strongly disagree) to 5 (strongly agree):**

|  | 1 | 2 | 3 | 4 | 5 | N/A |
| --- | --- | --- | --- | --- | --- | --- |
| This module is valuable to train *inexperienced* surgeons with regards to *robot assisted* procedures |  |  |  |  |  |  |
| This module is valuable to train *experienced* surgeons with regards to *robot assisted* procedures |  |  |  |  |  |  |
| This module is valuable to assess the skills of a trainee |  |  |  |  |  |  |

**The usability of the simulator**

**Rate on a scale of 1 (strongly disagree) to 5 (strongly agree):**

|  | 1 | 2 | 3 | 4 | 5 | N/A |
| --- | --- | --- | --- | --- | --- | --- |
| The instructions on how to use the simulator by the research assistant were clear |  |  |  |  |  |  |
| The simulator interface (its on-screen design) is user-friendly |  |  |  |  |  |  |
| The RobotiX VR simulator is an appealing training tool for this task |  |  |  |  |  |  |

**Task 3: Vaginal cuff closure**

Please rate the following statements on a scale of 1 to 5, meaning:

**1**: Strongly disagree **2**: Disagree **3**: Neutral **4**: Agree **5**: Strongly agree

**Realism**

**Rate on a scale of 1 (strongly disagree) to 5 (strongly agree):**

|  | 1 | 2 | 3 | 4 | 5 | N/A |
| --- | --- | --- | --- | --- | --- | --- |
| The on­screen response of the tools matched my actions |  |  |  |  |  |  |
| The physical effort to manipulate the graspers was sufficiently realistic |  |  |  |  |  |  |
| The representation of this task to mimic cuff closure in the clinical setting was sufficiently realistic |  |  |  |  |  |  |
| The behavior of the tissue was sufficiently realistic |  |  |  |  |  |  |
| The behavior of sutures running through the tissue was sufficiently realistic |  |  |  |  |  |  |

**The didactic value**

**Rate on a scale of 1 (strongly disagree) to 5 (strongly agree):**

|  | 1 | 2 | 3 | 4 | 5 | N/A |
| --- | --- | --- | --- | --- | --- | --- |
| This module is valuable to train *inexperienced* surgeons with regards to *robot assisted* procedures |  |  |  |  |  |  |
| This module is valuable to train *experienced* surgeons with regards to *robot assisted* procedures |  |  |  |  |  |  |
| This module is valuable to assess the skills of a trainee |  |  |  |  |  |  |

**The usability of the simulator**

**Rate on a scale of 1 (strongly disagree) to 5 (strongly agree):**

|  | 1 | 2 | 3 | 4 | 5 | N/A |
| --- | --- | --- | --- | --- | --- | --- |
| The instructions on how to use the simulator by the research assistant were clear |  |  |  |  |  |  |
| The simulator interface (its on-screen design) is user-friendly |  |  |  |  |  |  |
| The RobotiX VR simulator is an appealing training tool for this task |  |  |  |  |  |  |

Do you have any further remarks about the exercises or this study? Please state these below.

…………………………………………………………………………………………………………………………………………

…………………………………………………………………………………………………………………………………………

…………………………………………………………………………………………………………………………………………

**Thank you for your participation!**

**
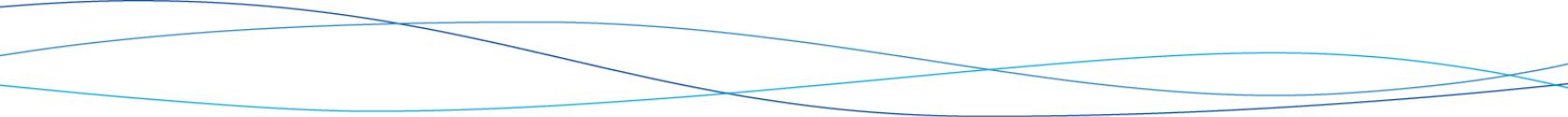
**
